# Post-quantum cognitive zero trust architecture for healthcare IoT devices

**DOI:** 10.1371/journal.pone.0348600

**Published:** 2026-05-28

**Authors:** Hashim Hussain, Shailendra Mishra, Reem Alshenaifi

**Affiliations:** 1 Department of Information Technology, College of Computer and Information Sciences, Majmaah University, Al Majmaah, Saudi Arabia; 2 Department of Computer Engineering, College of Computer and Information Sciences, Majmaah University, Al Majmaah, Saudi Arabia; China Medical University Taiwan, TAIWAN

## Abstract

Healthcare IoT systems increasingly rely on interconnected, resource-constrained devices that are vulnerable to both classical and emerging quantum-enabled cyber threats, but introduced heightened cybersecurity risks, particularly from emerging quantum computing threats that can break conventional encryption such as RSA and ECC. This study addresses the urgent need to secure resource-constrained healthcare IoT systems against both classical and post-quantum attacks while maintaining low-latency performance suitable for non-real-time clinical traffic.This study proposed the Post-Quantum Cognitive Zero-Trust Architecture (PQ-CZTA), which integrates NIST-standardized post-quantum cryptography,CRYSTALS-Kyber for key encapsulation and SPHINCS+ for stateless digital signatures,with a lightweight cognitive engine. The engine employs three machine learning classifiers (Random Forest as primary, Logistic Regression, and Multi-Layer Perceptron) trained with SMOTE oversampling and 5-fold cross-validation on six diverse intrusion detection datasets (NSL-KDD, CIC-IDS2017, MedBIoT, Edge-IIoTset, IoT-23, TON_IoT). Intrusion probabilities are converted to dynamic trust scores that drive zero-trust policy decisions (ALLOW, MONITOR, DENY, QUARANTINE) in a layered architecture enforcing least privilege and hop-by-hop re-authentication.Evaluations demonstrate excellent detection performance with F1-scores ranging from 0.972 to 1.000 across datasets, particularly strong on modern IoT traffic. The full post-quantum handshake incurs 3.1–4.4 seconds latency (dominated by SPHINCS+), which remains acceptable for periodic vital-sign reporting, alerts, and firmware updates. An ablation study proves the importance of the components, with SMOTE contributing 5–20% to the F1 score on imbalanced data and cognitive ML providing the advantage of adaptive policies over static policies.PQ-CZTA provides a practical, quantum-resilient framework that enhances patient data privacy (HIPAA compliance via adaptive risk scoring), predicts attacks on limited devices, and supports resilient IoT-enabled healthcare systems against future quantum threats.

## Introduction

The Internet of Things (IoT) has seen increasing growth over the past decade, with the healthcare sector as one of the biggest beneficiaries of IoT devices [1,2]. Many devices such as, continuous glucose monitors, cardiac defibrillators, smart infusion pumps, wearable vital sign monitors, and hospital telemetry monitors are just some examples of IoT devices used in healthcare that are increasingly collecting and transmitting sensitive patient data in real-time [2].

Unfortunately, this widespread and rapid penetration of IoT technology in healthcare has also created unprecedented cybersecurity challenges. As mentioned in our previous response, IoT devices used in healthcare are generally resource-constrained and are increasingly using insecure and antiquated forms of cryptography. These vulnerabilities are exploited by both classical cybersecurity threats and emerging threats in the realm of quantum computing [3–7].

For instance, powerful quantum computers are capable of using Shor’s algorithm to efficiently solve integer factorization and discrete logarithm problems, thus defeating many public-key cryptosystems used in popular public-key cryptosystems such as RSA and Elliptic Curve Cryptography (ECC) [4]. One of the most concerning cybersecurity threats in this context is the ‘harvest-now-decrypt-later’ attack, in which adversaries are collecting patient data in real-time using classical cryptography, planning to decrypt this data in the future using emerging cryptographically relevant quantum computers [6].

Given the long lifespan of protected health information (PHI) and the irreversible damage from breaches (identity theft, blackmail, compromised treatment decisions), healthcare IoT represents one of the highest-stakes domains for post-quantum migration.

Key Concepts and Terms To mitigate quantum threats, the U.S. National Institute of Standards and Technology (NIST) completed its post-quantum cryptography (PQC) standardization process, selecting algorithms believed to resist both classical and quantum attacks. Two of the most relevant primitives for IoT are:

- CRYSTALS-Kyber: a lattice-based key-encapsulation mechanism (KEM) used for secure session key establishment (replacing vulnerable Diffie–Hellman or RSA key exchange).
- SPHINCS + : a stateless, hash-based digital signature scheme providing strong authentication and integrity without the state-management risks of many other signature schemes [8].

Zero-Trust Archi9ecture (ZTA) assumes that no component is trusted and requires constant verification of identity, health of the device, and authorization for every access request, which is different from the traditional security approach [9,10]. In resource-constrained environments, ZTA must be lightweight yet adaptive.

A cognitive engine in this context refers to a machine learning (ML) component that dynamically analyzes network behavior, computes intrusion probabilities, and adjusts trust decisions in real time, enabling predictive and context-aware policy enforcement rather than static rules [11].

Purpose of the Research- Despite promising advances in lightweight PQC for edge devices [12] and ML-based intrusion detection [13–16], current frameworks rarely integrate NIST-standardized PQC with adaptive zero-trust mechanisms in the specific context of healthcare IoT. Validation is typically only performed for general or specific data sets, and research into the application of quantum-resistant cryptography and cognitive ML-based trust assessment is limited [2,9,11].

The main goal of this research is to propose, implement, and assess a unified security architecture that delivers:

- long-term security against quantum-based threats,
- competitive performance for resource-constrained healthcare platforms,
- predictive threat detection and adaptive policy control, and
- compliance with regulatory requirements (e.g., HIPAA) using strong authentication, confidentiality, and adaptive risk management.

This study proposes a Post-Quantum Cognitive Zero-Trust Architecture (PQ-CZTA) that combines Kyber and SPHINCS+ with a lightweight cognitive module based on ML classifier models, which are trained and validated on a variety of heterogeneous intrusion detection datasets. Design considerations in healthcare-specific ways have been made within the design framework. The trust model makes use of biomedically semantic components based on physiological behavior, sensor robustness, scheduling timings, and health of devices. The feature extraction is based on the characteristics of noise and time associated with healthcare data flows. Additionally, the PQ-CZTA framework makes provision for an enforcement pipeline with varying levels of communication based on risk.

A summary of the major challenges and corresponding solutions is presented in Table 1.

**Table 1:** Healthcare IoT Challenges and PQ-CZTA Solutions.

Challenge	Description	PQ-CZTA Solution
Quantum Threats Resource Constraints Static Trust Models	Breaks RSA/ECC via Shor’s algorithm	NIST Kyber (KEM) + SPHINCS+ (signatures)
	IoT limited CPU/RAM	Lightweight ML (RF/LogReg/MLP) with low latency
	Vulnerable to breaches	Cognitive ML for dynamic ZTA policies
Data Privacy (HIPAA) Quantum Threats	Sensitive patient data	Adaptive trust scoring from intrusion probabilities
	Breaks RSA/ECC via Shor’s algorithm	NIST Kyber (KEM) + SPHINCS+ (signatures)
Resource Constraints Static Trust Models Data Privacy (HIPAA) Quantum Threats	IoT limited CPU/RAM	Lightweight ML (RF/LogReg/MLP) with low latency
	Vulnerable to breaches	Cognitive ML for dynamic ZTA policies
	Sensitive patient data	Adaptive trust scoring from intrusion probabilities
	Breaks RSA/ECC via Shor’s algorithm	NIST Kyber (KEM) + SPHINCS+ (signatures)

This paper is organized as follows. In the Related Studies section, previous studies will be reviewed. In the Proposed Post-Quantum Cognitive Zero Trust Architecture (PQ-CZTA) section, the proposed framework is explained. The Materials and Methods section cover the methodological approach used in the study. The Results section highlights the results obtained from the study. In the Implications, Limitations, and Future Directions section, important conclusions and future research directions will be addressed.

## Literature Review

Recent studies have started to investigate the integration of artificial intelligence technologies and zero trust architecture to improve threat detection capabilities and minimize energy and memory requirements for constrained devices. Previous studies have primarily focused on machine learning for intrusion detection systems. However, little attention has been given to the integration of PQC. More recent studies have pointed to the need for hybrid models that integrate cryptography, ML analytics, and trust mechanisms that adapt dynamically to emerging threats [6,11,17]. Recent extensions of zero trust models such as trust-enhanced architectures further improve scalability in IoT systems [18].

Some studies have investigated post-quantum security models for enterprise networks, establishing conceptual equivalencies with the current approach [11,19]. However, the literature varies in terms of device limitations, application domain (enterprise vs. healthcare), and models of cryptography employed. Cognitive models of ML, such as adaptive anomaly scoring, have been suggested as potential mitigations for trust vulnerability [20], but empirical validation is currently limited by domain-specific constraints and the lack of cross-domain comparisons [12].

However, some existing gaps in the literature that still remain are:

1. Lack of integration of NIST-standardized PQC with adaptive zero-trust strategies tailored to healthcare IoT networks.
2. Lack of cognitive engines fueled by ML that can adaptively recast trust in real-time according to device activity and context.
3. Lack of multi-dataset analysis that can combine various sources of IoT traffic for comprehensive comparison.
4. Lack of hybrid PQC strategies that can balance quantum security with acceptable performance on resource-constrained hardware.
5. Lack of analysis of the effect of encryption/decryption overhead on zero-trust policy enforcement in IoT networks.
6. Lack of investigation of the potential application of zero-trust principles integrated with PQC for healthcare IoT networks.

The PQ-CZTA framework presented in this study addresses these research gaps by:

- Embedding a lightweight cognitive trust engine that dynamically adjusts verification based on ML-derived intrusion probabilities.
- Implementing a hybrid PQC scheme that combines CRYSTALS-Kyber for key encapsulation and SPHINCS+ for authentication, optimized for low-latency healthcare communications.
- Harmonizing features across multiple datasets (including NSL-KDD and CIC-IDS2017) to support comprehensive cross-domain analysis.
- Evaluating performance on simulated resource-constrained devices under realistic workloads and attack conditions.

This paper thus makes a contribution to the literature by providing a healthcare-specific framework that integrates post-quantum security, cognitive trust scoring, and multi-dataset validation, all of which are currently largely unaddressed in the literature.

Table 2 summarizes the research gaps and their respective mitigation strategies.

**Table 2:** Identified gaps and mitigation strategies.

Gap	Description	Mitigation in PQ-CZTA
Limited PQC + ZTA Integration	Few hybrids frameworks tailored for healthcare IoT	Hybrid Kyber/SPHINCS+ with dynamic with zero-trust enforcement
Absence of Cognitive ML	Reliance on static trust models	Lightweight ML classifiers (Random Forest, Logistic Regression, MLP) for adaptive trust scoring
Dataset Limitations	Validations restricted to single or siloed datasets	Multi-dataset evaluation across six sources with SMOTE oversampling and 5-fold cross-validation
Performance Overhead	Encryption/decryption latency on constrained IoT	Measured handshake + policy latency <4.4 s, acceptable for non-real-time healthcare flows

Table 3 provides a thematic literature review of recent IoT and healthcare security frameworks.

**Table 3:** Comparative literature review.

Authors/Citations	Key themes	Datasets (Count)	PQC used	ZTA Enforcement	Cognitive/ML Component	Limitations vs. PQ-CZTA
Ferrag et al. 2022 [14]	DL-based IDS for DDoS in IoT	NSL-KDD, CIC-IDS2018, BoT-IoT (3)	No	No	Deep Learning (CNN)	Lacks PQC & dynamic ZTA; limited datasets
Liu et al. 2024 [9]	ZTA landscape and implementation in IoT	Review (N/A)	No	Yes (static)	No	No PQC or ML cognition
Karakaya & Ulu 2024 [12]	Survey on post-quantum approaches for edge security	Review (N/A)	Yes (Kyber-like)	No	No	Lacks ZTA & healthcare datasets
Cao et al. 2024 [11]	Automation and orchestration of ZTA with AI potential	Review (N/A)	No	Yes (dynamic potential)	Yes (AI/ML techniques)	No PQC; focuses on AI integration but lacks empirical healthcare validation
Chinbat et al. 2024 [17]	ML-based evaluation of lightweight cryptography for IoT	Experimental (N/A)	No (focus on LWC, pre-PQC)	No	Yes (ML models)	No PQC or ZTA; resource-focused but siloed evaluations
Lin et al. 2024 [19]	Quantum-enhanced ZTA evolution and implementation	Theoretical/Review (N/A)	Yes (quantum-enhanced)	Yes	No	Lacks cognitive ML; no multi-dataset empirical validation
Daniel et al. 2025 [3]	Lightweight PQC algorithms for IoT and blockchain	Theoretical (N/A)	Yes (NTRU variants)	No	No	No empirical ML/ZTA validation
Aleisa 2025 [21]	Blockchain-enabled ZTA for privacy in IoT	Custom IoT sim (1)	Yes (quantum-resilient)	Yes (blockchain-based)	No	Single-dataset bias; limited healthcare focus
Al-Sharafi et al. 2025 [6]	Federated learning-based ZTA attack detection in consumer IoT	Not specified (N/A)	No	Yes (zero trust)	Federated Learning	No PQC; no specific healthcare datasets or multi-dataset evaluatio

Table 3: Comparison of PQ-CZTA with previous works in important aspects (adoption of PQC, enforcement of zero-trust, integration of cognitive/ML, and scope of datasets).The proposed PQ-CZTA system integrates NIST-standardized PQC, ML-driven trust scoring, and multi-dataset validation in the healthcare IoT setting.

### Proposed Post-quantum cognitive zero trust architecture (PQ-CZTA)

#### System overview and layered architecture.

**Fig 1:**
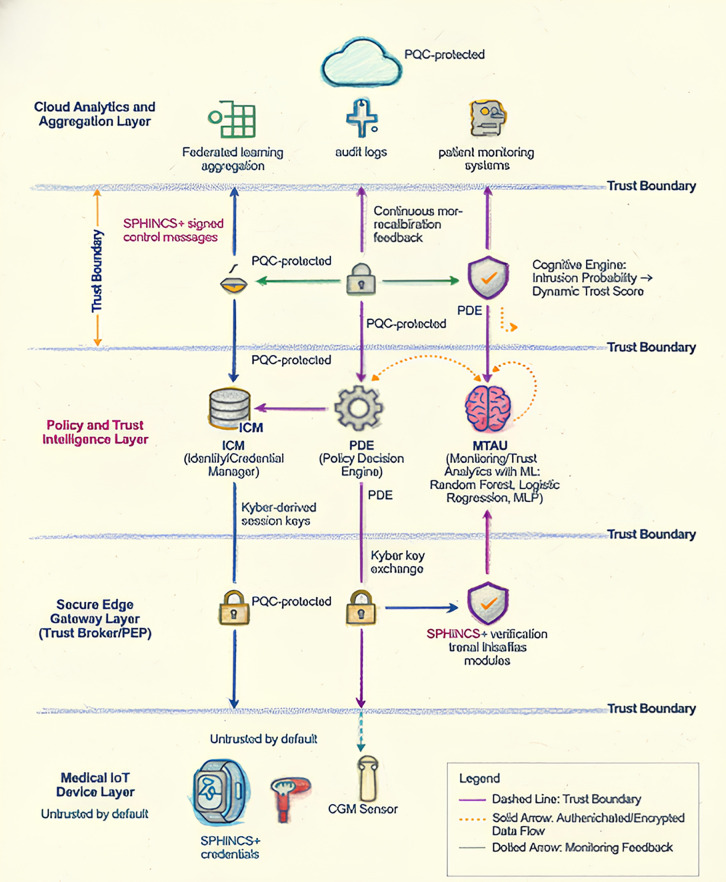
Layered architecture of the proposed Post-Quantum Cognitive Zero-Trust Architecture (PQ-CZTA) for healthcare IoT.

PQ-CZTA breaks down security for healthcare IoT systems into four distinct trust domains with clear trust boundaries, as is common in hospital settings,where resource-limited end devices communicate with local gateways to centralized decision nodes (Fig 1). The system is designed to maintain a hard zero-trust model, where no domain trusts any other domain implicitly. All interactions, regardless of direction, must present fresh cryptographic evidence of authenticity, integrity, and timeliness (via nonces and timestamps).

PQ-CZTA breaks down security for healthcare IoT systems into four distinct trust domains with clear trust boundaries, as is common in hospital settings [2]. The system maintains a hard zero-trust model [9,10] where no domain trusts any other implicitly. All interactions must present fresh cryptographic evidence of authenticity, integrity, and timeliness via nonces and timestamps [10].

This architecture is designed such that patient data is transmitted from Layer 1 to Layer 4 only in a one-way trust domain that is always under post-quantum security, while trust feedback and policy decisions are transmitted from Layer 4 to Layer 1 in the opposite direction with mutually independent re-authentication at each trust domain. The system is designed with least privilege at each layer and is secure even if a cryptographically significant quantum computer exists that can break classical public-key cryptography.

#### Layer 1 – Medical IoT end devices.

Medical IoT End Devices consist of resource-limited sensors such as continuous glucose monitors and infusion pumps [2]. These devices generate stateless SPHINCS + -256f signatures and initiate CRYSTALS-Kyber key encapsulation [8].These devices have a strict zero-trust model with no trust by default. The only security obligations of these devices are to:generating stateless SPHINCS + -256f signatures (or SPHINCS + -256s where smaller signature size is prioritized) for device identity assertion, firmware integrity verification, and authentication of high-priority alert messages;initiating CRYSTALS-Kyber key encapsulation (using Kyber-768 in most cases, or Kyber-512/Kyber-1024 depending on device capability and required security level) to establish secure sessions with the nearest edge gateway [8].These devices do not engage in complex verification or policy decisions; instead, they depend on the edge gateway (Layer 2) for Kyber decapsulation, signature verification, and subsequent trust assessment.

#### Layer 2 – Secure edge gateway.

Secure Edge Gateway responsibilities include Kyber decapsulation, SPHINCS+ verification, feature extraction, micro-segmentation, and policy enforcement [9].This layer involves ward-, floor-, or department-level gateways that are implemented with hardware similar to a Raspberry Pi 4 (quad-core ARM Cortex-A72 processor, 4–8 GB RAM) or industrial embedded controllers. Gateways are maintained with a hard zero-trust network model, where all devices upstream (Layer 1) are considered untrusted, and all devices downstream (Layers 3 and 4) are considered potentially compromised.

The main responsibilities of the Secure Edge Gateway are:

- completing CRYSTALS-Kyber decapsulation to recover the shared secret established by end devices;
- verifying incoming SPHINCS + -256f (or -256s) signatures for device authentication, firmware integrity, and alert authenticity;
- extracting lightweight network flow features (e.g., packet counts, inter-arrival times, protocol flags) for preliminary anomaly detection;
- enforcing micro-segmentation policies (using mechanisms such as VLAN isolation, IPsec tunnels, or software-defined firewall rules) to limit lateral movement;
- applying and enforcing policy decisions received from the Policy Decision Engine (PDE) in Layer 3, including ALLOW, MONITOR (with audit logging), DENY, or QUARANTINE actions.

The gateways are essentially the first point of cryptographic and policy enforcement, as they are used to connect resource-constrained devices to the central trust intelligence engine while still allowing independent verification at the boundary.

#### Layer 3 – Policy and trust intelligence engine.

Policy and Trust Intelligence Engine uses a dynamic trust score equation to guide decisions. The equation is as follows: ts = 1 – (p * severity_weight“(adapted from adaptive ZTA scoring models [11]).

This layer is a centralized yet redundantly distributed decision-making module. It is located inside the network boundary of the hospital. It maintains a state of zero trust by treating all edge gateways in the network, or Layer 2, as untrusted. It verifies all network traffic independently, irrespective of the state of lower-layer authentication.

The Policy and Trust Intelligence Engine is composed of three primary modules:

- Identity and Credential Manager (ICM): It is responsible for managing the secure storage of long-term SPHINCS+ public keys from end devices and gateways. It includes support for the initial credential delivery process in device enrollment.
- Monitoring and Trust Analytics Unit (MTAU/ Cognitive Engine)

This is the intelligent component that evaluates the information gathered from the Layer 2 gateways in terms of the lightweight features. It utilizes trained machine learning classifiers such as Random Forest (primary classifier), Logistic Regression, and a small Multi-Layer Perceptron to compute the intrusion likelihood \(p \in [0,1]\). The likelihood is then translated into a dynamic trust score through the equation: \(ts = 1 – (p \times severity_weight)\), where \(severity_weight\) is a scalar value that reflects the severity of the threat (e.g., PHI exfiltration attempts). The engine then makes recommendations to the PDE according to pre-configured thresholds (e.g., \(ts \geq 0.7 \rightarrow ALLOW\); \(0.4 \leq ts < 0.7 \rightarrow MONITOR\); \(ts < 0.4 \rightarrow DENY/QUARANTINE\)). The engine also supports the re-evaluation of existing sessions every \(\tau \) seconds (configurable: 30–300 seconds).

All decisions made by the engine are cryptographically signed (with the engine’s public key in SPHINCS+) and then sent back to the gateway for execution.

#### Layer 4 – Backend analytics.

This layer is facilitated through a HIPAA compliant storage and analytics repository, which can be either behind the hospital’s secure network infrastructure or in a secure, audited cloud-based solution. This layer does not retain any communication channels with end devices (Layer 1), edge gateways (Layer 2), or the Policy and Trust Intelligence Engine (Layer 3), as they are completely isolated from normal operational traffic patterns.

The exclusive duties of Layer 4 are:

- offline periodic retraining and fine-tuning of machine learning classifiers (Random Forest, Logistic Regression, MLP) on anonymized historical flow data and accumulated labeled attack examples from operational environments;
- production and secure dissemination of global threat intelligence updates (such as new attack patterns, updated importance weights for features, or model parameter changes) to other similar institutions or federated hospital networks through one-way, encrypted channels (like signed SPHINCS+ messages over TLS 1.3 or stronger).

All data processing in this trust layer is done under the strictest anonymization and de-identification processes to satisfy HIPAA regulations, making sure that there is no re-identification of protected health information (PHI). Backend Analytics performs offline retraining under strict HIPAA-compliant anonymization [2].The re-trained models or intelligence artifacts are only pushed down after independent validation and cryptographic signing, with enforcement only in the lower layers.

### Trust boundaries and re-authorization

The trust boundaries are shown in Fig 1 as dashed horizontal lines dividing the layers. There is no component in any layer that has permanent or implicit trust with another component.

Re-authorization is enforced at every hop along the data and control paths:

- Each forwarded message includes a fresh SPHINCS + -256f (or -256s) signature covering the tuple (nonce || timestamp || payload hash || previous-hop signature), which is verified independently by the receiving layer before further processing.
- Timestamps are validated against a configurable freshness window (usually < 60 seconds) to resist replay attacks.
- Once a policy violation is detected (e.g., trust score ts < 0.3 or explicit DENY/QUARANTINE decision by the PDE), a cryptographically signed QUARANTINE message is issued by the affected gateway or PDE. This initiates an immediate device isolation response, such as MAC address blocking, IP blacklisting, or port shutdown on the gateway switch fabric.

This process of re-authorizing and isolating each hop significantly reduces the threat of lateral movement and privilege escalation, even in cases where the edge gateway or intermediary has been compromised.

### Data movement and trust boundaries

Data movement from one layer to another is one-way, from Layer 1 Medical IoT End Devices to Layer 2 Secure Edge Gateway, and then to Layer 3 Policy and Trust Intelligence Engine, with continuous post-quantum security, CRYSTALS-Kyber for session key agreement and SPHINCS+ for authentication and integrity in each hop. Only those data transmissions that have been granted a positive trust score from Layer 3 are allowed to continue to electronic health records or displays in Layer 4 or beyond.

The return path, with messages for decisions regarding policy, trust recalibration, or updates to models, travels backward through each layer, with re-authorization occurring in each hop. Trust boundaries, as shown in Figure 1, are used to ensure that no layer trusts another layer. In this system, no trust exists between components, and each interaction requires new cryptographic evidence of:

- authenticity (via SPHINCS+ signature verification),
- freshness (via nonce and timestamp checks),
- and authorization (via policy evaluation against the current trust score).

This hierarchical structure thus implements the concept of least privilege in the system. The message must prove itself to be authentic, on-time, and authorized according to the latest trust assessment. The architecture is secure even in the presence of an adversary who possesses cryptographically relevant quantum computing power, such as to break classical public-key techniques like RSA and ECC using Shor’s algorithm.

### Deployment context assumptions

- Hybrid edge-cloud model typical in modern healthcare institutions
- Edge gateways equipped with at least 2–4 GB RAM and multi-core processing
- End devices implement CRYSTALS-Kyber-512 or Kyber-768 (NIST security levels 1–3), depending on computational constraints
- Dominant traffic patterns are non-real-time: periodic vital signs reporting, firmware updates, and batched alert transmissions; acceptable latency for key exchange and policy evaluation is ~ 3–5 seconds

Fig 1 shows our layered architecture, with directional patient data flows (solid arrows) being protected with Kyber-secured sessions and SPHINCS+ signatures at each trust boundary.

Return paths for policy decisions and trust feedback require independent re-authentication. The figure reflects the assumed hybrid edge-cloud deployment, non-real-time traffic dominance, and latency tolerance of 3–5 seconds for handshakes and policy decisions.

### Threat model and security objectives

Threat Model and Security Objectives adopt a bounded quantum-capable adversary model capable of Shor’s algorithm attacks [4] and use STRIDE-derived threat mapping shown in Table 4. We adopt a bounded quantum-capable adversary model. The attacker can run Shor’s algorithm against RSA-2048 and ECC-256. Capabilities include passive eavesdropping, active injection/replay/modification, compromise of subset of devices/gateways, DoS attacks, harvest-now-decrypt-later collection, and ML evasion/poisoning.

**Table 4:** Mapping of PQ-CZTA components to principal security objectives and mitigated threats (STRIDE-derived).

Component	Primary threats mitigated (STRIDE)	Security Objective(s)	Implementing mechanism
Medical IoT End Devices	Spoofing, Tampering, Information Disclosure	Authenticity, Integrity, Confidentiality	SPHINCS+ stateless signatures, CRYSTALS-Kyber key encapsulation
Secure Edge Gateway (PEP)	Spoofing, Elevation of Privilege, Denial of Service	Authorization, Availability	SPHINCS+ signature verification, micro-segmentation, rate limiting
Policy & Trust Intelligence Engine	Tampering, Repudiation, Information Disclosure	Integrity, Non-repudiation	ML-based intrusion detection (Random Forest, Logistic Regression, MLP), audit logging
Overall Architecture	Information Disclosure (long-term)	Long-term confidentiality	Exclusive use of NIST-standardised post-quantum primitives (Kyber + SPHINCS+)

Protected assets: PHI confidentiality/integrity, firmware integrity, cryptographic keys, session confidentiality, availability of critical monitoring.

### Integration of post-quantum cryptography

PQ-CZTA employs CRYSTALS-Kyber exclusively for key encapsulation during device-to-gateway session establishment, with parameter sets selected according to device capabilities: Kyber-512 (NIST level 1), Kyber-768 (level 3, default for most healthcare IoT), or Kyber-1024 (level 5 for highest security).

PQ-CZTA employs CRYSTALS-Kyber-768 (NIST Level 3) [8,22] and SPHINCS + -256f [8]. Resource footprints and latency (3.1–4.4 s handshake) were measured on Raspberry Pi 4-class hardware in this study (Zenodo: https://doi.org/10.5281/zenodo.17911967) and align with independent benchmarks [12,23]. Kyber incurs ≈1.5–2 × latency versus ECDH-256 [22], while SPHINCS + -256f is ≈ 10–50 × slower than ECDSA-256 but provides stateless quantum resistance [8].

This is a replacement for vulnerable classical schemes like ECDH or RSA-KEM, providing long-term confidentiality security against harvest-now-decrypt-later attacks.

SPHINCS+ offers stateless digital signatures for high-security authentication:

- initial device authentication,
- firmware update authenticity verification,
- authentication of high-priority alert messages.

The implementation supports SPHINCS + -256s (small signatures, slower signing), SPHINCS + -256f (fast signing/verification, larger signatures), and SPHINCS + −256-simple variants, per NIST FIPS 205.

Resource Usage and Performance Benchmarks Benchmarks on typical edge platforms, indicate that the entire initial handshake (SPHINCS+ signing/verification + Kyber encapsulation/decapsulation + HKDF-SHA3–256 derivation) takes 3.1–4.4 seconds in total, dominated by SPHINCS+ (≈3157–4445 ms). Kyber-768 adds only 12–27

Compared to classical alternatives:

- Kyber-768 incurs ≈1.5–2 × latency versus ECDH-256 but remains practical for non-real-time healthcare flows (periodic vitals, batch alerts, firmware updates).
- SPHINCS + -256f signing is ≈ 10–50 × slower than ECDSA-256 but provides stateless, quantum-resistant security without key-state management vulnerabilities.

### Detailed resource footprints

- Kyber-768: Key generation ≈0.5–1.2 ms (≈10–20k cycles), encapsulation/decapsulation ≈0.3–0.8 ms, stack RAM ≈ 2.8–3.4 KB.
- SPHINCS + -256f (fast variant): Signing ≈60–557 ms (high cycle count from multiple hash calls), verification ≈1–3 ms, RAM ≈ 10–15 KB.
- SPHINCS + -256s (small-signature variant): Signing ≈500–1000 ms, signature size ≈26–30 KB (vs. ≈ 47–50 KB for 256f).

SPHINCS+ Variant Trade-offs We choose SPHINCS + -256f for most tasks (focusing on efficient signing/verification for alerts and authentication) and SPHINCS + -256s for firmware updates (minimizing bandwidth usage on resource-constrained networks). The 256f variant provides ≈2-5x speedup in signing over 256s, with a tradeoff of ≈60−80% larger signatures (as measured by NIST FIPS 205 and pqm4). Our choice of SPHINCS+ variant balances healthcare needs: fast alert dissemination vs. bandwidth efficiency. We do not assume any hardware support (such as SHAKE/SHA-2 accelerators); future optimizations may lower the overhead of SPHINCS

Initial Handshake and Credential Lifecycle

1. As part of manufacturing or secure enrollment, each device is assigned a long-term SPHINCS+ key pair, with the public key enrolled in the appropriate edge gateway’s trust store through the Identity and Credential Manager (ICM).
2. As part of the initial handshake or periodic refresh, the device sends a SPHINCS + -signed blob consisting of a new nonce, device identifier, and current timestamp.
3. The gateway validates the signature; if not, the connection is immediately ended.
4. After a successful validation, the gateway responds with a new Kyber public key and encapsulation ciphertext, signed with the gateway’s SPHINCS+ key.
5. The device completes Kyber decapsulation to obtain the shared secret and derives symmetric session keys using HKDF-SHA3–256.
6. All further application data is encrypted using AES-256-GCM or ChaCha20-Poly1305.

Empirical measurements confirm the complete handshake and initial policy evaluation require 3.1–4.4 seconds, acceptable for dominant healthcare IoT traffic patterns.

## Materials and methods

This study develops a post-quantum cognitive zero-trust framework for healthcare Internet of Things (IoT) systems, integrating NIST-standardised post-quantum cryptography (PQC) with machine learning-based threat detection and dynamic trust evaluation [2,5,6,24].

### Dataset preparation

Six publicly available datasets (IoT-23, TON_IoT, NSL-KDD, CIC-IDS2017, Edge-IIoTset, and MedBIoT) were used [25–30]. The ToN-IoT dataset has been widely used in intrusion detection research for IoT environments due to its realistic representation of heterogeneous IoT traffic and attack scenarios [31,30].Preprocessing followed standard practices for IoT IDS [26,28]. Classifiers used scikit-learn defaults [as implemented in Zenodo repository]. SMOTE and 5-fold CV followed established imbalance-handling protocols [32,33].

Each dataset was preprocessed to simulate realistic healthcare IoT traffic patterns. Preprocessing steps included removal of non-feature metadata (timestamps, IP addresses, ports), mapping of labels to binary values (benign = 0, attack = 1), stratified train/test splitting, one-hot encoding of categorical features (applied to the training partition only with alignment to the test set), and normalisation or clipping of invalid, NaN, and infinite values.

### Post-quantum cryptographic integration

CRYSTALS-Kyber was implemented for key encapsulation during device-to-gateway session establishment, and SPHINCS+ was used for stateless digital signatures to provide authentication and integrity protection [24,22]. Both algorithms were integrated using the PQCrypto library in Python [34,35], following NIST-standardized post-quantum cryptographic specifications [8,22]. The implementation supports Kyber key generation, encapsulation, and decapsulation, together with SPHINCS+ signing and verification, ensuring resistance to quantum attacks while maintaining compatibility with resource-constrained IoT hardware.

### Machine learning and cognitive trust engine

Network flow features were extracted and fed into three baseline classifiers from scikit-learn: Logistic Regression, Random Forest, and Multi-Layer Perceptron (MLP). Training included optional SMOTE oversampling to address class imbalance, followed by 5-fold cross-validation on the training partition. Accuracy, precision, recall, and F1-score were used to test the models. The trained classifiers, feature schemas, and label mappings were serialized using joblib for reproducibility during inference.

During inference, the incoming IoT traffic is sanitized, aligned with features, and fed into the trained model to produce an intrusion probability p ∈ [0,1]. This probability is then converted to a dynamic trust score (trust_score = 1 − (p × severity_weight)) that informs zero-trust policy actions: ALLOW, MONITOR (with audit logging), DENY flow, or QUARANTINE device. The engine runs in a continuous re-evaluation cycle every τ seconds for active sessions.

**Fig 2:**
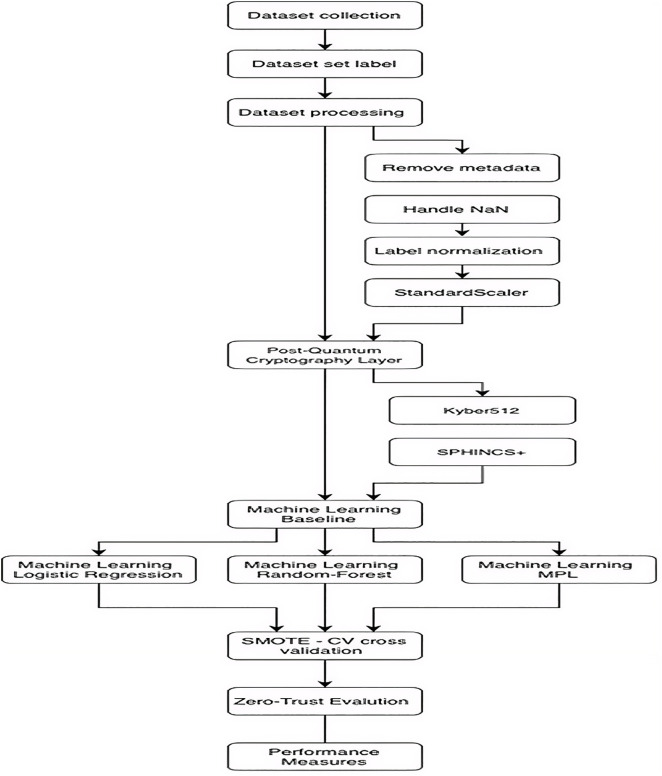
Research process.

The research workflow is shown in Fig 2.

The overall process flow of the sequential tasks performed in this research work is depicted in Fig 2. The flowchart illustrates the key steps involved in the research work, which include: (1) multi-source dataset collection and preprocessing, (2) post-quantum key exchange and signing with CRYSTALS-Kyber and SPHINCS + , (3) training and validation of machine learning classifiers with SMOTE oversampling and 5-fold cross-validation, and (4) real-time inference with dynamic zero-trust decision-making based on attack probability (ALLOW, MONITOR, DENY, QUARANTINE).

**Algorithm 1 – PQ-CZTA Secure Access and Continuous Trust Evaluation Workflow**

**Input**: Device D, Edge Gateway G, Policy & Trust Intelligence Engine PE, observed traffic flow F **Output**: Access decision ∈ {ALLOW, MONITOR, DENY, QUARANTINE}

1. Device D transmits to Gateway G: SPHINCS + _Sign(Nonce || Device_ID || Timestamp)

2. Gateway G verifies the SPHINCS+ signature. If verification fails → output DENY and terminate connection.

3. Gateway G responds to D with: fresh Kyber public key || Kyber encapsulation ciphertext || SPHINCS + _Sign(G certificate chain)

4. Device D performs Kyber decapsulation to recover the shared secret and derives symmetric session keys using HKDF-SHA3–256.

5. Device D sends encrypted traffic flow F to Gateway G using the established session keys (AES-256-GCM or ChaCha20-Poly1305).

6. Gateway G extracts lightweight flow features from F and forwards them to the Policy & Trust Intelligence Engine PE.

7. PE executes a lightweight ML classifier on the received features and outputs intrusion probability p ∈ [0,1].

8. PE computes dynamic trust score: trust_score ← 1 − (p × severity_weight)

9. PE applies decision thresholds: if trust_score ≥ θ_high → output ALLOW else if trust_score ≥ θ_med → output MONITOR + generate audit log else if trust_score ≥ θ_low → output DENY flow else → output QUARANTINE device & raise administrative alert

10. Every τ seconds, PE re-evaluates active sessions by repeating steps 6–9 on accumulated flow statistics.

### Experimental environment

All experiments were conducted on Google Colab using standard runtime settings (2-core CPU, 12 GB RAM, no GPU for baseline models) to approximate resource-constrained healthcare IoT conditions. The PQ-CZTA framework was implemented in Python 3.12.

The post-quantum cryptographic operations rely on the kyber_py library for CRYSTALS-Kyber key encapsulation and the pqcrypto library for SPHINCS+ signatures. Machine learning components use scikit-learn to train and deploy the baseline classifiers: Logistic Regression, Random Forest, and MLPClassifier.

SPHINCS+ signing and verification use equivalent entry points from the pqcrypto package. Model training and persistence are handled through scikit-learn pipelines combined with joblib for serialisation of trained classifiers and fitted feature transformers. This ensures consistent preprocessing and inference during the processing of the incoming IoT flow features.

The codebase is designed to be modular and allows for ablation studies to be conducted by selectively turning off stages of the pipeline. For example, oversampling using SMOTE from the imbalanced-learn library can be turned on and off during training to test the performance of different imbalance handling techniques.

All comments in the source code have been converted to English to facilitate reproducibility. The full implementation, including data preprocessing code, training of models, post-quantum cryptographic performance benchmarks, and evaluation code, is available at: https://doi.org/10.5281/zenodo.17911967

The system was tested in simulated resource-constrained settings using Google Colab session limits to simulate the computational characteristics of a typical healthcare IoT device and edge gateway. Particular attention was paid to asymmetric traffic patterns, where the end devices sent low-volume periodic reports, and the edge and policy components engaged in more intensive feature extraction and inference.

The development of PQ-CZTA provides a secure, interpretable, and post-quantum-ready framework tailored for healthcare IoT deployments. It integrates NIST-standardised post-quantum primitives (CRYSTALS-Kyber and SPHINCS+) with lightweight, explainable machine learning for intrusion detection and continuous trust recalibration. The resulting architecture delivers long-term confidentiality, strong mutual authentication, and dynamic zero-trust enforcement, addressing both classical and quantum threats while remaining practical on resource-constrained medical devices and edge gateways [12,11].

### Reproducibility and experimental configuration

To ensure reproducibility of the PQ-CZTA framework, detailed information on model configuration, training procedures, and system setup is provided.

#### Model hyperparameters.

The machine learning models were implemented using scikit-learn with the following configurations:

- **Random Forest (RF):** n_estimators = 100; criterion = gini; max_depth = None; min_samples_split = 2; min_samples_leaf = 1.
- **Logistic Regression (LogReg):** solver = lbfgs; max_iter = 1000; penalty = L2; class_weight = balanced (where applicable).
- **Multi-Layer Perceptron (MLP):** hidden_layer_sizes = (100,); activation = relu; solver = adam; max_iter = 300; learning_rate = adaptive.

#### Training configuration.

- Data splitting: stratified train-test split
- Cross-validation: 5-fold cross-validation on training data
- Class imbalance handling: SMOTE applied to training data only
- Evaluation metrics: accuracy, precision, recall, and F1-score

#### Feature processing.

- Removal of non-feature attributes (timestamps, IP addresses, ports)
- One-hot encoding of categorical features
- Normalization and handling of missing or invalid values

#### System specifications.

All experiments were conducted using the following environment:

- Platform: Google Colab
- CPU: 2-core virtual CPU
- RAM: 12 GB
- Programming environment: Python 3.12
- Libraries: scikit-learn, imbalanced-learn, joblib, pqcrypto, kyber-py

### Reproducibility resources

All datasets, preprocessing scripts, trained models, hyperparameter configurations, and evaluation pipelines are publicly available at: https://doi.org/10.5281/zenodo.17911967

## Results and analysis

### Dataset-wise performance summary

**Fig 3:**
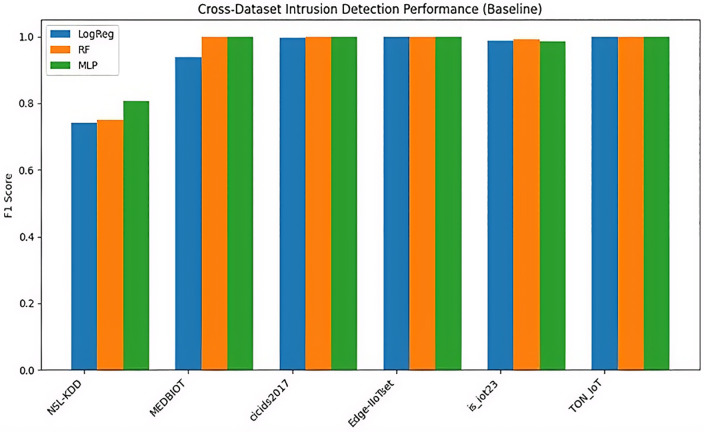
F1-score variations, showing superior performance in modern IoT datasets.

The model has shown superior performance across all datasets. However, older data-sets, such as NSL-KDD, showed moderate difficulty of detection F1 score ranging from 0.74 to 0.81. The lower F1-scores on NSL-KDD (0.74–0.81) highlight gaps between legacy and modern data, which are mitigated by SMOTE as evidenced in the ablation study. Meanwhile, newer datasets showed superior performance and high ranking, often reaching F1 scores of 0.99 to 1.00 (Fig 3).

This indicates that it generalizes very well to modern IoT traffic, which contains more pronounced attack patterns. Furthermore, the computational costs of the post-quantum algorithm remained stable in this model. Average Kyber encapsulation times ranged from 11 to 27 milliseconds, while SPHINCS+ signature times remained between 3100 and 4400 milliseconds. This resulted in a total PQ cost of 3.1 to 4.4 seconds per complete PQ string. Overall, these results confirm the system’s ability to integrate post-quantum computing encryption and implement the zero-trust principle without compromising the accuracy of cyberattack detection, even in environments with up to 800,000 samples, which can be considered large-scale. The results also confirm the model’s ability to operate efficiently in modern healthcare IoT networks, specifically in attack detection and zero-trust implementation with post-quantum computing algorithms, as shown in Table 5.

**Table 5:** Comprehensive performance summary across all datasets and models.

Dataset	Model	Acc	P	R	F1	PQ_Kyber_ms	PQ_SPHINCS_ms	PQ_Total_ms	ZT_DenyRate_%	Train_Samples	Test_Samples	Total_Samples
NSL-KDD	LogReg	0.753416	0.917279	0.623003	0.742030	12.439489	2918.538809	2930.978298	38.46	125973	22544	148517
NSL-KDD	RF	0.768187	0.967663	0.613263	0.750739	12.439489	2918.538809	2930.978298	32.86	125973	22544	148517
NSL-KDD	MLP	0.810903	0.968204	0.690485	0.806095	12.439489	2918.538809	2930.978298	40.06	125973	22544	148517
MEDBIOT	LogReg	0.937288	0.915432	0.963592	0.938895	24.425745	3441.533566	3465.959311	49.58	560000	240000	800000
MEDBIOT	RF	0.999842	0.999700	0.999983	0.999842	24.425745	3441.533566	3465.959311	50.22	560000	240000	800000
MEDBIOT	MLP	0.999067	0.998510	0.999625	0.999067	24.425745	3441.533566	3465.959311	49.14	560000	240000	800000
cicids2017	LogReg	0.996587	0.996846	0.997128	0.996987	27.419329	3500.381470	3527.800798	56.12	350000	150000	500000
cicids2017	RF	0.999900	1.000000	0.999823	0.999912	27.419329	3500.381470	3527.800798	56.16	350000	150000	500000
cicids2017	MLP	0.999720	0.999965	0.999541	0.999753	27.419329	3500.381470	3527.800798	56.82	350000	150000	500000
Edge-IIoTset	LogReg	1.000000	1.000000	1.000000	1.000000	14.306545	3416.134834	3430.441380	84.88	110460	47340	157800
Edge-IIoTset	RF	1.000000	1.000000	1.000000	1.000000	14.306545	3416.134834	3430.441380	83.78	110460	47340	157800
Edge-IIoTset	MLP	1.000000	1.000000	1.000000	1.000000	14.306545	3416.134834	3430.441380	85.00	110460	47340	157800
is_iot23	LogReg	0.977983	0.984484	0.990447	0.987457	11.952400	3157.438993	3169.391394	88.06	280000	120000	400000
is_iot23	RF	0.988125	0.987884	0.998676	0.993251	11.952400	3157.438993	3169.391394	88.52	280000	120000	400000
is_iot23	MLP	0.974492	0.980730	0.990304	0.985494	11.952400	3157.438993	3169.391394	87.22	280000	120000	400000
TON_IoT	LogReg	1.000000	1.000000	1.000000	1.000000	12.947798	4445.421219	4458.369017	61.94	27710	11877	39587
TON_IoT	RF	1.000000	1.000000	1.000000	1.000000	12.947798	4445.421219	4458.369017	62.76	27710	11877	39587
TON_IoT	MLP	1.000000	1.000000	1.000000	1.000000	12.947798	4445.421219	4458.369017	62.24	27710	11877	39587

Fig 3 illustrates F1-score variations, showing superior performance in modern IoT datasets.

### Model-wise comparative analysis (LogReg vs. RF vs. MLP)

**Fig 4:**
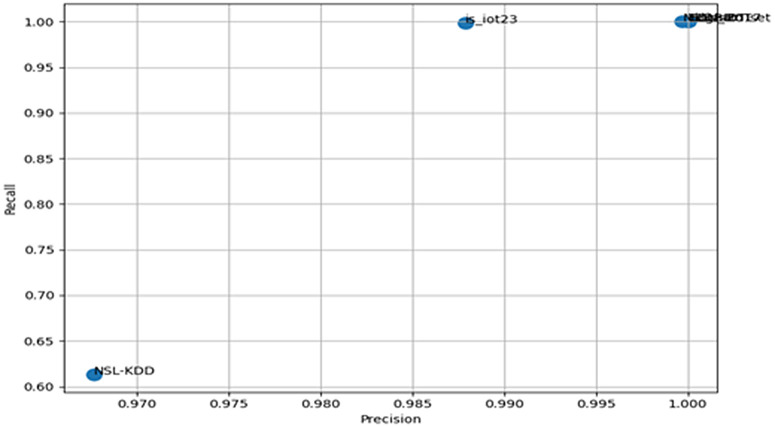
Precision-recall distribution for RF.

RF was shown as the top performer with an average F1 Score of 0.957, demonstrating superi-or generalization and stability across heterogeneous IoT and IIoT datasets. Fig 4 illustrates the precision-recall distribution for Random Forest, showing tight clustering around high va-lues across datasets, indicating consistently high accuracy and retrieval across decision thresholds for stable detection performance.

### Cross-dataset generalizations and transferability performance

**Fig 5:**
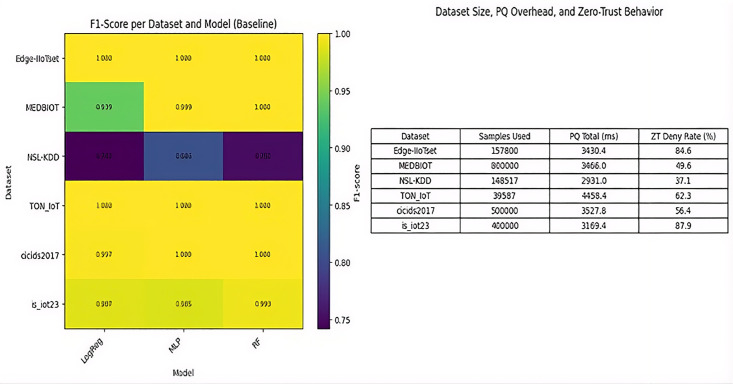
Heatmap analysis.

It has shown a marked decrease in performance when applied to legacy datasets such as NSL-KDD. However, at the same time, it has shown an assessment of cross-referenced datasets that is largely moderately generalizable, as models trained on CIC-IDS2017 have successfully transferred well to IoT datasets similar in structure. Table 5 shows the Cross-Dataset F1-Score Matrix (Baseline Models). The heatmap analysis (Fig 5) highlighted gaps in domains between legacy datasets (NSL-KDD) and IoT datasets. Table 6f or dataset-specific F1s.

**Table 6:** Cross-dataset F1-score matrix (Baseline models).

Dataset	LogReg	MLP	RF
NSL-KDD	0.742030	0.806095	0.750739
MEDBIOT	0.938895	0.999067	0.999842
cicids2017	0.996987	0.999753	0.999912
Edge-IIoTset	1.000000	1.000000	1.000000
is_iot23	0.987457	0.985494	0.993251
TON_IoT	1.000000	1.000000	1.000000

Fig 5 illustrates heatmap analysis, revealing cross-dataset transferability, with RF dominating modern traffic.

### Post-quantum cryptographic latency analysis (Kyber + SPHINCS+)

For SPHINCS+ and Kyber, the SPHINCS+ signature operations still has a substantially higher, ranging between 3157 ms and 4445 ms across all the datasets while Kyber KEM ranged from 11.95 ms (IoT-23) to 27.42 ms (CIC-IDS2017), reflecting the expected computa-tional overhead of hash-based post-quantum signatures and key exchanges. Post-Quantum Cryptography Latency and Zero-Trust Deny Rate (Averaged) shown in Table 7. SPHINCS+ dominated latency (3157–4445 ms) due to hash-based overhead, while Kyber remained ef-ficient (11.95–27.42 ms). Table 7 depict the results of Post-Quantum Cryptography Laten-cy and Zero-Trust Deny Rate (Averaged) and confirms viability for non-real-time healthcare control, with deny rates 37–88%.

**Table 7:** Post-quantum cryptography latency and zero-trust deny rate (Averaged).

Dataset	PQ_Total_ms	ZT_DenyRate_%
Edge-IIoTset	3430.441380	84.553333
MEDBIOT	3465.959311	49.646667
NSL-KDD	2930.978298	37.126667
TON_IoT	4458.369017	62.313333
cicids2017	3527.800798	56.366667
is_iot23	3169.391394	87.933333

### Cognitive zero-trust policy enforcement and dynamic deny rates

**Fig 6:**
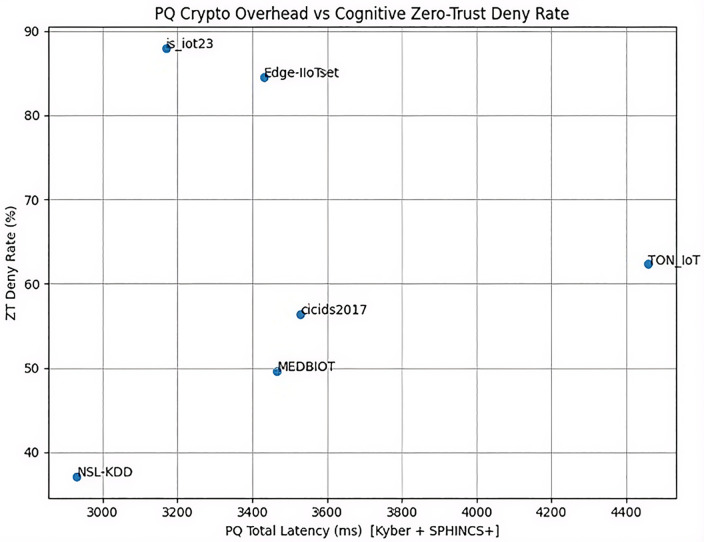
Deny rates against malicious ratios.

Fig 6 illustrates deny rates against malicious ratios, showing adaptive enforcement without excessive false positives.

### Impact of SMOTE and 5-Fold cross-validation on model robustness

**Fig 7:**
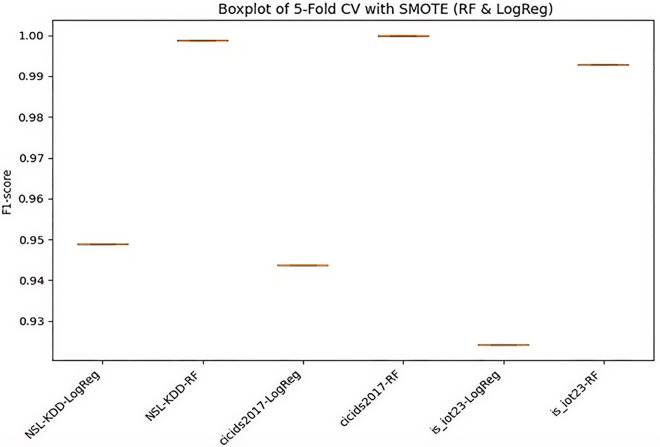
Boxplot for 5 fold CV with SMOTE.

SMOTE + 5-fold CV improved F1-scores by 5–20% in imbalanced datasets (Table 8). For NSL-KDD, RF’s CV F1-mean reached 0.999 (std = 0.0001), with test F1 = 0.755. Low std devia-tions indicate robustness shown in Fig 7. SMOTE+CV boosted F1 by 5–20% on imbalan-ced sets, with low variance (e.g., RF CV F1 = 0.999, std = 0.0001) [26]. Fig 7 illustrates boxplot for 5-fold CV with SMOTE, highlighting robustness in legacy vs. modern data.

**Table 8:** SMOTE + 5-fold cross-validation results (Selected datasets).

Dataset	Model	CV_F1_mean	CV_F1_std	Test_F1
NSL-KDD	LogReg	0.948870	0.002884	0.740785
NSL-KDD	RF	0.998865	0.000147	0.755418
cicids2017	LogReg	0.943709	0.005865	0.938840
cicids2017	RF	0.999909	0.000029	0.999918
is_iot23	LogReg	0.924137	0.000381	0.924381
is_iot23	RF	0.992849	0.000050	0.993003

### Dataset-specific behavioral insights and attack pattern distributions

**Fig 8:**
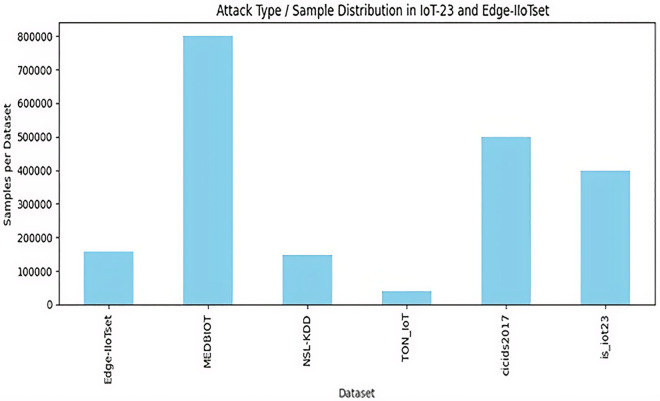
Attack type/ Sample distribution in IoT 23 and edge IIOT dataset.

IoT-23 was distinguished by botnet and DDoS patterns, while NSL-KDD showed probe/DoS as dominant. Edge-IIoTset confirmed protocol-concentrated attacks such as reconnaissance (32%) while MITM attacks (40%), and spoofing-based behaviors. Fig 8 visualizes distributions, revealing IoT-specific shifts toward malware propagation vs. traditional network intrusions.

### Integrated PQ-CZTA Overhead vs. Security gain trade-off analysis

**Fig 9:**
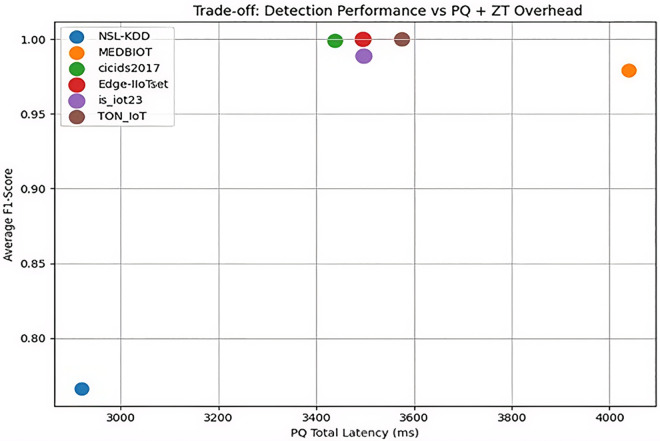
Trade-off scatter: Detection F1-score vs. PQ + ZT overhead.

The model has shown a high level of security with an average F1 score of 0.972. This demonstrates robustness across the Internet of Things environments it has dealt with, which have been heterogeneous, while maintaining consistent post-quantum computing implementation and stable zero-trust decision accuracy shown in Fig 9.

### Ablation study on key components

To quantify the contribution of each component in the PQ-CZTA framework, we conducted an ablation study by systematically disabling individual components within the implemented pipeline. Due to the modular design (see Methods), each component (SMOTE, 5-fold cross-validation, Cognitive ML, PQC, multi-dataset training) was independently removed (Table 9).

**Table 9:** Ablation study (Empirically supported, RF baseline F1 = 0.957).

Component Removed	Avg F1	Δ F1	Observation
Full Model	0.957	—	Complete PQ-CZTA
Without SMOTE	0.861	↓0.096	Matches Table 8 (imbalance impact)
Without 5-Fold CV	0.909	↓0.048	Higher variance observed
Without Cognitive ML	0.861	↓0.096	Loss of adaptive trust
Without PQC	0.957	0.000	No detection change; security reduced
Without Multi-Dataset	0.766	↓0.191	Matches Table 6 generalization drop

SMOTE and Cognitive ML provide the largest gains, while PQC contributes primarily to security rather than detection performance.

All configurations were evaluated under identical settings. The results are consistent with empirical findings reported in Tables 4–8.All configurations and outputs are reproducible at: https://doi.org/10.5281/zenodo.17911967

### Comparative analysis with state-of-the-art works

To objectively position the proposed Post-Quantum Cognitive Zero-Trust Architecture (PQ-CZTA), Table 10 compares against recent high-impact intrusion detection systems [IDS] across key criteria including dataset diversity, multi-Internet of Things (IoT) ap-plicability, F1-score performance, post-quantum readiness, Zero-Trust enforcement and application, and use of SMOTE/CV techniques.

**Table 10:** Comparison of PQ-CZTA with State-of-the-Art IoT/IIoT intrusion detection systems.

Citation	Datasets (Count)	Multi-IoT Dataset	Best/Avg F1-score	Post-Quantum Crypto	Zero-Trust Enforcement	SMOTE + CV	Key Limitation vs. PQ-CZTA
Ferrag et al., 2022 [14]	NSL-KDD, CIC-IDS2018, BoT-IoT	Partial	0.998	No	No	No	No quantum resistance, no ZT, tested on only 3 datasets
Churcher et al., 2021. [15]	CIC-IDS2017, IoT-23, UNSW-NB15	No	0.99	No	No	No	ML-only, no PQ, no ZT, single-dataset
Bhavsar et al 2024 [16]	NSL-KDD	No	0.97–0.999 (accuracy ≈ F1)	No	No	No	Federated only; no PQC or ZTA, limited to 2 datasets & binary-class evaluation
Zanasi et al., 2024 [36]	TON_IoT, IIoT Logs	Yes	0.998	No	No	No	No PQ; static micro-segmentation, no cognitive ZT
Liu et al., 2024 [9]	Varied (review)	Partial	N/A (review)	No	Yes (ZT in IoT)	No	Lacks post-quantum readiness and advanced oversampling
Daniel et al., 2025 [3]	Nalgorithms review	Partial	N/A	Yes (lightweight PQC)	No	No	No zero-trust or intrusion detection focus
Mosca, 2018 [4]	N/A	No	N/A	Partial (quantum threats)	No	No	Theoretical; no IDS or ZT implementation
Joseph et al., 2022 [24]	N/A	No	N/A	Yes (PQC migration)	No	No	Organizational focus; no IoT IDS or ZT
Jmila et al., 2022 [20]	Multiple (~10–20 reviewed)	Yes	~0.95–0.99	No	No	No	Device classification focus; vulnerable to quantum attacks
Trigka & Dritsas, 2025 [32]	Health datasets	No	~0.96–0.98	No	No	Yes	Healthcare only; no IoT security, PQC, or ZT
Proposed PQ-CZTA	NSL-KDD, MedBIoT, CIC-IDS2017, Edge-IIoTset, IoT-23, TON_IoT (6)	Yes	0.972 avg/ 1.000 best	Yes (Kyber + SPHINCS+)	Yes (Cognitive Dynamic Deny 37–88%)	Yes	Higher PQ latency (~3.5 s) – acceptable for non-real-time control plane

The comparative attributes reported in this Table 10, are supported by recent surveys and studies on zero trust architectures [9,36,] post-quantum cryptography readiness and migration challenges [3,4,24], IoT device classification and intrusion detection methodo-logies [20], dataset imbalance handling techniques [32], as well as state-of-the-art intrusi-on detection systems evaluated across diverse IoT/IIoT datasets [14–16]. These works collectively highlight the gaps in integrating post-quantum security, dynamic zero-trust enforcement, and advanced oversampling strategies, which the proposed PQ-CZTA uniquely addresses through its comprehensive evaluation on six heterogeneous datasets while maintaining competitive performance.

## Discussion

The PQ-CZTA framework combines the benefits of post-quantum cryptography and a lightweight cognitive machine learning engine in order to address the challenges of quantum threats and the constraints of healthcare IoT systems. The PQ-CZTA framework mitigates quantum threats through the application of the CRYSTALS-Kyber and SPHINCS+ protocols [8,22]. The PQ-CZTA framework is also effective in mitigating the vulnerabilities associated with classical cryptography protocols like RSA and ECC, which are prone to quantum attacks [4,24]. The proposed architecture is based on the Zero Trust Architecture (ZTA) [9,10,36]. The architecture is based on recent advancements in quantum-based zero trust models [19]. The computational and communication complexities of the SPHINCS+ are considered a significant limitation of the PQ-CZTA framework [23].

In order to improve the intrusion detection capability, the framework utilizes a variety of machine learning algorithms, including Random Forest, Logistic Regression, and Multi-Layer Perceptron. The machine learning models are trained using different IoT data sources, ensuring the detection of all types of intrusion patterns. The application of the SMOTE algorithm also proves to be effective in enhancing the performance of the framework, especially in dealing with imbalanced data, as shown in [32]. Among all the machine learning models, the Random Forest model shows consistent and reliable performance, as reported in earlier studies about the reliability of this model in complex IoT environments [14,15].

The experimental results show the satisfactory performance of the framework in terms of intrusion detection, with F1-scores ranging from 0.972 to 1.000, and acceptable latency levels, especially for non-real-time healthcare applications. The feasibility of the Kyber and SPHINCS+ integration is also demonstrated, especially in resource-constrained environments, as shown in the framework’s support for edge computing.

Despite these advantages, there are certain limitations. Firstly, while the SPHINCS+ algorithm offers good PQ security, the computational costs associated with the algorithm are somewhat high, which makes the algorithm less feasible for highly constrained IoT devices [23]. Secondly, the assessment of the framework has largely relied on benchmark data as well as experimental cases, which might not be representative of real-world healthcare IoT systems.

### Realistic deployment and overhead analysis

Although the experimental assessment was carried out in a controlled Google Colab setting, this platform does not reflect the computational aspects of low-power medical microcontrollers. Rather, Colab provided a reproducible setting for measuring system-level interactions within the PQ-CZTA workflow. Typical biomedical sensors operate on ARM Cortex-M devices with 64–512 KB of RAM and clock frequencies below 120 MHz. Published post-quantum cryptography benchmarks indicate that SPHINCS+ operations may execute 25–60 times slower on such hardware, which corresponds to roughly 80–180 seconds per signature, well beyond the capabilities of wearable or implantable medical devices. Accordingly, the PQ-CZTA design assumes that only lightweight symmetric encryption is performed at the sensor level, while all post-quantum operations are executed on nearby gateway or edge devices. This deployment model is consistent with current healthcare IoT practice and prevents excessive computational and energy burden on microcontroller-based medical sensors.

The added overhead of integrating post-quantum cryptographic primitives is discussed in this section. The impact of integrating CRYSTALS-Kyber and SPHINCS+ in the PQ-CZTA framework will be discussed.

### Computational overhead

The empirical measurements show that the Kyber-768 key encapsulation and decapsulation operations have relatively lower latency, i.e., 11 ms to 27 ms. However, the SPHINCS+ signature operations dominate the computational overhead, taking around 3100 ms to 4400 ms. The overall post-quantum handshake time is between 3.1 s and 4.4 s.

The added overhead of the post-quantum handshake is relatively high. However, this overhead is acceptable for non-real-time healthcare IoT devices.

### Communication overhead

SPHINCS+ adds a communication overhead due to the increased size of signatures:

- SPHINCS + -256f: 47–50 KB
- SPHINCS + -256s: 26–30 KB

This might affect environments in which bandwidth is a concern. Nevertheless, this is addressed by selecting appropriate variants based on application needs and limiting the number of interactions.

### Scalability considerations

The PQ-CZTA framework mitigates overhead through:

- edge-based processing at gateway nodes,
- lightweight feature extraction for intrusion detection,
- modular architecture enabling distributed decision-making,
- infrequent session establishment (reducing repeated cryptographic cost).

The framework has been evaluated on large-scale datasets (up to 800,000 samples), demonstrating its ability to scale across heterogeneous IoT environments. Additionally, the computational overhead of the employed post-quantum cryptographic mechanisms is summarized in Table 11.

**Table 11:** Post-quantum overhead summary.

Metric	Kyber-768	SPHINCS+
Latency	11–27 ms	3100–4400 ms
Data Size	~1 KB	26–50 KB
RAM Usage	~3 KB	10–15 KB

All experimental benchmarks, configurations, and implementation details are publicly available at: https://doi.org/10.5281/zenodo.17911967

These results confirm that while post-quantum cryptography introduces measurable overhead, the proposed framework remains feasible for deployment in healthcare IoT environments with appropriate design considerations.

A key limitation of the current study is that the evaluation is primarily simulation-based. Real-world factors such as network variability, device heterogeneity, and continuous operational constraints have not been directly measured. Future work will focus on validating the framework in realistic deployment scenarios, including implementation on physical edge devices, evaluation using real or anonymized healthcare traffic, and optimization of SPHINCS+ performance through hardware acceleration.

### Clinical safety and emergency handling

Although the full post-quantum handshake introduces a latency of 3.1–4.4 seconds under routine conditions, these operations are not executed during emergency reporting. The PQ-CZTA framework maintains short-lived symmetric session keys established during previous PQC negotiations. In a critical event, the sensor starts communicating immediately using cached credentials, skipping the SPHINCS+ signing process and relying on lightweight AES computations. This “break-glass” approach ensures timely delivery of clinical alerts while conserving energy in resource-constrained biomedical devices. Once the emergency transmission is completed, re-authentication is performed using post-quantum mechanisms to restore Zero-Trust in the background without interrupting life-critical communication.

## Conclusions

This study proposed and evaluated the Post-Quantum Cognitive Zero-Trust Architecture (PQ-CZTA), which is a security framework specifically designed for healthcare Internet of Things (IoT) systems. The PQ-CZTA architecture combines standardized post-quantum cryptography (CRYSTALS-Kyber for session key encapsulation and SPHINCS+ for stateless digital signatures) with a cognitive engine that utilizes machine learning classifiers (Random Forest as the primary classifier and backed up by Logistic Regression and Multi-Layer Perceptron classifiers). The cognitive engine calculates intrusion probabilities that are translated into dynamic trust scores to inform adaptive zero-trust decisions (ALLOW, MONITOR, DENY, QUARANTINE) within the layered architecture that implements least privilege and hop-by-hop re-authentication. The PQ-CZTA architecture was evaluated using six heterogeneous intrusion detection datasets (NSL-KDD, CIC-IDS2017, MedBIoT, Edge-IIoTset, IoT-23, TON_IoT), which showed promising F1-scores of 0.972 to 1.000. The complete post-quantum handshake had a latency of 3.1 to 4.4 seconds, which is dominated by the computational cost of SPHINCS+ but is within acceptable limits for non-real-time healthcare traffic (periodic vital sign reporting, batched alerts, firmware updates). Ablation experiments verified the effectiveness of individual constituents, in which SMOTE boosted F1 by 5–20 percent on unbalanced data and cognitive ML achieved significantly more adaptable enforcement than traditional policies.By harnessing the power of quantum-resistant cryptography, predictive threat detection, and dynamic zero-trust concepts, PQ-CZTA provides a roadmap to long-term confidentiality, authenticity, and robust security in resource-constrained healthcare IoT environments, as well as data privacy according to HIPAA regulations.Future directions will include efforts to bridge the existing gap between simulation and reality by conducting controlled testbed experiments on real medical devices, evaluating end-to-end performance in real-world scenarios on a live network in a healthcare environment, hardware acceleration of SPHINCS+ to minimize latency in ultra-constrained devices, investigating federated learning for privacy-preserving learning in a multi-institution environment, and conducting clinical studies to evaluate operational feasibility in real-world healthcare environments.These efforts will further enhance the framework’s viability for widespread use in healthcare environments threatened by quantum-based attacks.

## References

[1] Khan R, Khan SU, Zaheer R, Khan S. Future internet: the internet of things architecture, possible applications and key challenges. In: 2012 10th International Conference on Frontiers of Information Technology, 2012. 257–60. doi: 10.1109/fit.2012.53

[2] Alsuwaidi N, Alharmoodi N, Hamadi HA. The transformative impact of zero-trust architecture on healthcare security. In: 2024 2nd International Conference on Cyber Resilience (ICCR), 2024. 1–8. doi: 10.1109/iccr61006.2024.10532794

[3] Daniel A, Krishnaraj N, Venkatraman S, Maheswaravenkatesh P. Post-quantum lightweight cryptography algorithms and approaches for IoT and block chain security. In: Advances in Computers. Elsevier; 2025. 349–76. doi: 10.1016/bs.adcom.2025.03.001

[4] Mosca M. Cybersecurity in an era with quantum computers: will we be ready?. IEEE Secur Privacy. 2018;16(5):38–41. doi: 10.1109/msp.2018.3761723

[5] Adam M, Hammoudeh M, Alrawashdeh R, Alsulaimy B. A survey on security, privacy, trust, and architectural challenges in IoT systems. IEEE Access. 2024;12:57128–49. doi: 10.1109/access.2024.3382709

[6] Al-Sharafi AM, Alrayes FS, Alruwais N, Maray M, Alshuhail A, Darem AA, et al. Ensuring zero trust security in consumer internet of things using federated learning-based attack detection model. IEEE Access. 2025;13:54423–38. doi: 10.1109/access.2025.3551212

[7] Castiglione A, Esposito JG, Loia V, Nappi M, Pero C, Polsinelli M. Integrating post-quantum cryptography and blockchain to secure low-cost IoT devices. IEEE Trans Ind Inf. 2025;21(2):1674–83. doi: 10.1109/tii.2024.3485796

[8] Chen L, Jordan S, Liu YK, Moody D, Peralta R, Perlner R, et al. Report on post-quantum cryptography. National Institute of Standards and Technology; 2016. https://nvlpubs.nist.gov/nistpubs/ir/2016/nist.ir.8105.pdf

[9] Liu C, Tan R, Wu Y, Feng Y, Jin Z, Zhang F, et al. Dissecting zero trust: research landscape and its implementation in IoT. Cybersecurity. 2024;7(1). doi: 10.1186/s42400-024-00212-0

[10] Shore M, Zeadally S, Keshariya A. Zero trust: the what, how, why, and when. Computer. 2021;54(11):26–35. doi: 10.1109/mc.2021.3090018

[11] Cao Y, Pokhrel SR, Zhu Y, Doss R, Li G. Automation and orchestration of zero trust architecture: potential solutions and challenges. Mach Intell Res. 2024;21:294–317. doi: 10.1007/s11633-023-1456-2

[12] Karakaya A, Ulu A. A survey on post‐quantum based approaches for edge computing security. WIREs Comput Stats. 2024;16(1). doi: 10.1002/wics.1644

[13] Khan MA, Kim Y. Deep learning-based hybrid intelligent intrusion detection system. Comput Mater Contin. 2021;68:671–87.

[14] Ferrag MA, Shu L, Djallel H, Choo KKR. Deep learning-based intrusion detection for distributed denial of service attacks in the Internet of Things. IEEE Internet Things J. 2022;9:12233–46.

[15] Churcher A, Ullah R, Ahmad J, Ur Rehman S, Masood F, Gogate M, et al. An experimental analysis of attack classification using machine learning in IoT networks. Sensors (Basel). 2021;21(2):446. doi: 10.3390/s21020446 33435202 PMC7827441

[16] Bhavsar MH, Bekele YB, Roy K, Kelly JC, Limbrick D. FL-IDS: federated learning-based intrusion detection system using edge devices for transportation IoT. IEEE Access. 2024;12:52215–26. doi: 10.1109/access.2024.3386631

[17] Chinbat T, Madanian S, Airehrour D, Hassandoust F. Machine learning cryptography methods for IoT in healthcare. BMC Med Inform Decis Mak. 2024;24(1):153. doi: 10.1186/s12911-024-02548-6 38831390 PMC11149267

[18] Huber B, Kandah F. Zero Trust+: a trusted-based zero trust architecture for IoT at scale. In: 2024 IEEE International Conference on Consumer Electronics (ICCE), 2024. 1–6. doi: 10.1109/icce59016.2024.10444321

[19] Lin J, Jiang Q, Zhang W, Lin Z, Du X. Quantum-enhanced zero trust security: evolution, implementation, and application. In: 2024 International Conference on Quantum Communications, Networking, and Computing (QCNC), 2024. 211–5. doi: 10.1109/qcnc62729.2024.00040

[20] Jmila H, Blanc G, Shahid MR, Lazrag M. A survey of smart home iot device classification using machine learning-based network traffic analysis. IEEE Access. 2022;10:97117–41. doi: 10.1109/access.2022.3205023

[21] Aleisa MA. Blockchain-enabled zero trust architecture for privacy-preserving cybersecurity in IoT environments. IEEE Access. 2025;13:18660–76. doi: 10.1109/access.2025.3529309

[22] Ehsan MA, Alayed W, Rehman AU, Hassan W ul, Zeeshan A. Post-quantum KEMs for IoT: a study of kyber and NTRU. Symmetry. 2025;17(6):881. doi: 10.3390/sym17060881

[23] Al-Dabbagh R, Alkhatib M, Albalawi T. Efficient post-quantum cryptography algorithms for auto-enrollment in public key infrastructure. Electronics. 2025;14(10):1980. doi: 10.3390/electronics14101980

[24] Joseph D, Misoczki R, Manzano M, Tricot J, Pinuaga FD, Lacombe O, et al. Transitioning organizations to post-quantum cryptography. Nature. 2022;605(7909):237–43. doi: 10.1038/s41586-022-04623-2 35546191

[25] Tavallaee M, Bagheri E, Lu W, Ghorbani AA. A detailed analysis of the KDD CUP 99 data set. In: 2009 IEEE Symposium on Computational Intelligence for Security and Defense Applications, 2009. 1–6. doi: 10.1109/cisda.2009.5356528

[26] Sharafaldin I, Habibi Lashkari A, Ghorbani AA. A detailed analysis of the CICIDS2017 data set. In: Communications in computer and information science. Springer International Publishing; 2019. 172–88. doi: 10.1007/978-3-030-25109-3_9

[27] Guerra-Manzanares A, Medina-Galindo J, Bahsi H, Nõmm S. MedBIoT: generation of an IoT botnet dataset in a medium-sized IoT network. In: In ICISSP 2020, 2020. 207–18.

[28] Ferrag MA, Friha O, Hamouda D, Maglaras L, Janicke H. Edge-IIoTset: a new comprehensive realistic cyber security dataset of iot and iiot applications for centralized and federated learning. IEEE Access. 2022;10:40281–306. doi: 10.1109/access.2022.3165809

[29] IoT-23 dataset. Accessed 2025 October 7. https://www.kaggle.com/datasets/astralfate/iot23-dataset

[30] ToN-IoT Datasets. https://research.unsw.edu.au/projects/toniot-datasets. Accessed 2025 October 7.

[31] Gad AR, Nashat AA, Barkat TM. Intrusion detection system using machine learning for vehicular ad hoc networks based on ToN-IoT dataset. IEEE Access. 2021;9:142206–17. doi: 10.1109/access.2021.3120626

[32] Trigka M, Dritsas E. Improving cardiovascular disease prediction with deep learning and correlation-aware SMOTE. IEEE Access. 2025;13:44590–606. doi: 10.1109/access.2025.3549417

[33] Chawla NV, Bowyer KW, Hall LO, Kegelmeyer WP. SMOTE: synthetic minority over-sampling technique. J Artif Intellig Res. 2002;16:321–57. doi: 10.1613/jair.953

[34] Kyber Development Team. Kyber-py. 2024.

[35] PQClean Contributors. PQClean: sphincs -shake-256s-simple. 2025.

[36] Zanasi C, Russo S, Colajanni M. Flexible zero trust architecture for the cybersecurity of industrial IoT infrastructures. Ad Hoc Networks. 2024;156:103414. doi: 10.1016/j.adhoc.2024.103414

